# Acral Plantar Melanoma Mimicking a Diabetic Foot Ulcer in a Nonagenarian: A Case Report

**DOI:** 10.7759/cureus.107644

**Published:** 2026-04-24

**Authors:** Gerardo Bascuñan, Monserrat Cendoya, Diego Guarda, Priscilla Marquez

**Affiliations:** 1 General Practice, Hospital Familiar y Comunitario de Carahue, Carahue, CHL; 2 Dermatology, Universidad de Santiago de Chile, Santiago, CHL; 3 General Practice, Clínica Andes Salud, Puerto Montt, CHL; 4 General Practice, Universidad Andres Bello, Santiago, CHL

**Keywords:** acral melanoma, biopsy, chronic wound, diabetic foot ulcer, elderly, misdiagnosis, plantar melanoma

## Abstract

Acral melanoma on the plantar surface may be clinically overlooked and can resemble common causes of plantar ulceration. In patients with diabetes, chronic plantar lesions are often managed as diabetic foot ulcers, which can delay consideration of alternative diagnoses. We report the case of a very elderly woman with diabetes whose plantar lesion was initially treated as a diabetic foot ulcer but later evolved with features concerning for malignancy. Histopathology confirmed deeply invasive ulcerated acral melanoma. This case highlights the importance of maintaining suspicion for malignant mimics in non-healing plantar lesions and supports early biopsy when a presumed diabetic foot ulcer behaves atypically or fails to improve with appropriate care.

## Introduction

Acral melanocytic lesions can be diagnostically challenging because plantar lesions may mimic benign, inflammatory, traumatic, or pressure-related processes and may not be routinely inspected during clinical encounters [1]. Acral melanoma is a subtype of cutaneous melanoma arising on glabrous skin of acral sites, including the palms, soles, and nail apparatus. Unlike most melanomas arising on chronically sun-exposed skin, acral melanoma develops in anatomically distinct locations, may show different clinicopathologic and molecular features, and can clinically overlap not only with benign acral melanocytic lesions but also with hyperkeratotic, traumatic, inflammatory, or ulcerative conditions [2,3]. Epidemiologically, acral melanoma is uncommon overall but accounts for a proportionally higher share of melanoma cases among individuals of Asian, Hispanic, and African ancestry, partly because ultraviolet-associated melanoma is less prevalent in these populations. In contrast to sun-exposed cutaneous melanomas, ultraviolet radiation appears to play a lesser etiologic role, whereas chronic mechanical stress or repeated trauma has been proposed as a potential contributing factor in some studies. Clinically, acral melanoma is frequently diagnosed at greater Breslow thickness and a more advanced stage, largely related to delayed recognition and anatomically concealed sites [2,3]. Contemporary reviews emphasize that acral and nail melanomas continue to be affected by delayed recognition and that clinical morphology can overlap with several non-malignant acral conditions, contributing to late-stage presentation [2]. Reviews focused on acral lentiginous melanoma further underscore that acral melanoma differs from sun-exposed cutaneous melanoma in epidemiology, etiologic factors, and clinicopathologic behavior, reinforcing the need for careful assessment of suspicious plantar lesions [3].

In patients with diabetes, a plantar melanoma may be initially managed as a diabetic foot ulcer, contributing to diagnostic delay. Multiple reports have described malignant melanoma misdiagnosed as a diabetic foot ulcer, illustrating how the diabetic-foot framework can obscure malignant mimics [4,5]. Additional case reports similarly describe plantar melanoma first treated as ulcer disease before biopsy established the diagnosis [6,7]. A systematic review of reported malignancies initially misdiagnosed as diabetic foot ulcers found that skin cancers, including melanoma, can present as chronic ulcers and highlighted the importance of biopsy in refractory or clinically atypical ulcers [8]. Such diagnostic delay is clinically relevant because it may result in greater Breslow thickness, a more advanced stage at presentation, and more limited therapeutic options at the time of diagnosis [8]. From a management standpoint, reviews addressing acral melanoma highlight that treatment decisions, especially in older adults with comorbidity and frailty, must be individualized, balancing oncologic benefit with functional status and goals of care [9]. In this context, we present a case of an elderly patient with diabetes in whom a plantar lesion initially treated as a diabetic foot ulcer was ultimately diagnosed as deeply invasive acral melanoma.

## Case presentation

**Figure 1:**
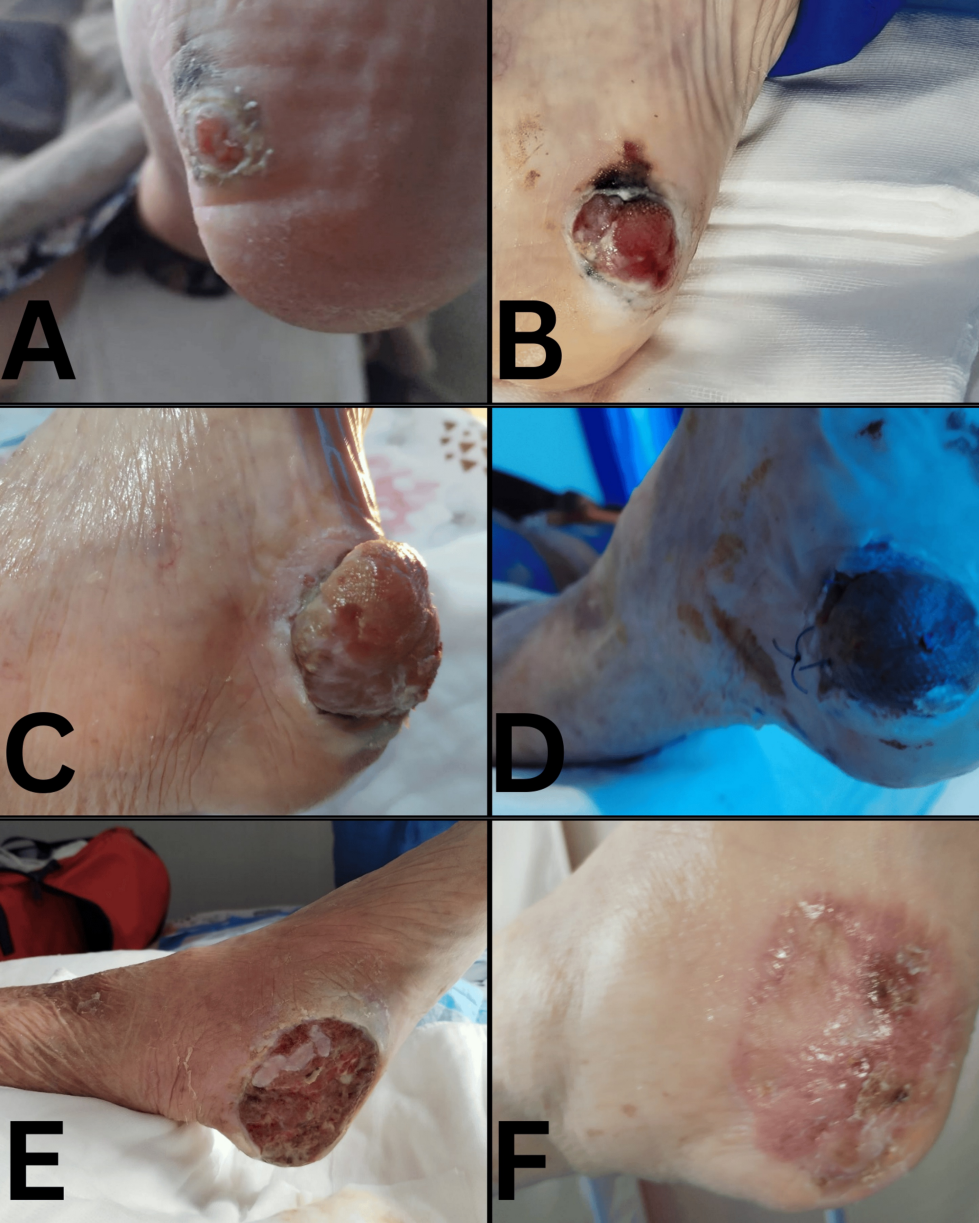
Clinical evolution of the left plantar lesion over approximately 10 months (A–F) Images are presented in chronological order, from initial presentation to late postoperative follow-up over an approximately 10-month period. Figure 1A: Initial lesion (initially interpreted as diabetic foot ulcer): Small plantar ulceration with a moist erythematous granulating base, surrounded by a hyperkeratotic/callus-like rim and perilesional maceration; focal brown-black pigmentation adjacent to one margin. The overall appearance can mimic a neuropathic/pressure-related diabetic ulcer. Figure 1B: Progression under wound care (enlarging ulcer with peripheral pigment): Larger ulcer with exuberant granulation tissue (“beefy-red” appearance), with areas of yellow-white fibrin/slough, a hyperkeratotic border, and persistent focal brown-black pigment at/near the margin, suggesting an atypical ulcer pattern. Figure 1C: Exophytic/nodular phase (clinical suspicion for malignancy): Exophytic nodulo-tumoral mass arising from the plantar heel with a lobulated/fungating contour, erythematous friable surface, and crust/exudate, with a surrounding hyperkeratotic collar. This morphology is atypical for a straightforward diabetic foot ulcer and is clinically compatible with a malignant process. Figure 1D: Post-surgical stage (reconstruction with sutures/graft): Immediate/early postoperative appearance with sutures in situ and a darkened covered wound surface consistent with graft/dressing changes; perilesional skin shows antiseptic staining and postoperative changes. Figure 1E: Complicated healing after excision (ulceration/slough): Large oval postoperative ulcer with granulation tissue, yellow fibrinous slough, and erythematous margins, compatible with delayed healing/partial graft loss after wide excision. Figure 1F: Later follow-up (partial re-epithelialization): Healing plantar area with partial re-epithelialization, residual erythematous plaque, superficial erosions, and serocrust, consistent with ongoing wound remodeling under conservative management.

A 92-year-old woman with a medical history of type 2 diabetes mellitus, arterial hypertension, and advanced dementia, with no known personal history of melanoma, non-melanoma skin cancer, or other malignancy, no relevant family history of melanoma or cutaneous malignancy, and no other clinically suspicious simultaneous skin lesions documented during the available evaluations, developed a lesion on the plantar surface of the left foot. The lesion evolved progressively over the following months, from a small ulcerative plantar lesion to a nodular exophytic tumor before definitive diagnosis. At symptom onset, the lesion was clinically interpreted as a diabetic foot ulcer and was managed with local wound care (Figure 1A). 

Over the following weeks, despite continued dressings, the lesion progressively enlarged. Clinically, it evolved into a deeper ulcer with exuberant granulation tissue and a peripheral hyperkeratotic/callus-like rim, with focal pigmentary change near one margin (Figure 1B). With ongoing progression, the lesion developed a prominent exophytic/nodular component with a friable surface, crusting, and serous to fibrinous exudate, prompting surgical documentation of tumoral suspicion and escalation to tissue diagnosis (Figure 1C).

After several months of progressive enlargement and transformation into an exophytic nodular lesion, an incisional biopsy was performed to obtain a tissue diagnosis. Histopathology demonstrated ulcerated malignant melanoma, acral subtype, infiltrating at least into the deep reticular dermis (Clark level IV), with Breslow thickness ≥5 mm and mitotic index 2/mm². Tumor-infiltrating lymphocytes (TILs) were absent. Lymphovascular invasion, perineural invasion, regression, satellitosis, and residual nevus were not identified. Immunohistochemistry showed positivity for S-100 protein, supporting melanocytic differentiation. Pathologic staging was reported as pT4b according to the American Joint Committee on Cancer (AJCC) 8^th^ edition staging system [10].

Subsequently, the patient underwent definitive surgical management with wide local excision and skin graft reconstruction (Figure 1D). The excisional specimen demonstrated ulcerated acral-fusocellular melanoma with deep infiltration reaching the hypodermis and dense fibroconnective tissue consistent with fascia (Clark level V), with Breslow thickness 19 mm and mitotic index 6/mm². TILs remained absent, and lymphovascular invasion, perineural invasion, satellitosis, regression, and residual nevus were again not identified. Margins were reported free, including a deep margin of 9 mm from the tumor. Final staging again corresponded to pT4b (AJCC 8^th^ edition [10]).

The incisional and excisional biopsy results are summarized in Table 1.

**Table 1:** Histopathological comparison between incisional and excisional specimens The incisional biopsy demonstrated ulcerated acral melanoma infiltrating at least to the deep reticular dermis, whereas the excisional specimen confirmed deeper invasion into the hypodermis and fascia, with increased Breslow thickness and mitotic index.

Feature	Incisional biopsy	Excisional specimen
Site	Left heel/plantar (incisional)	Left plantar resection
Subtype	Acral melanoma	Acral–fusocellular melanoma
Ulceration	Present	Present
Clark level	IV	V
Breslow thickness	≥5 mm	19 mm
Mitotic index	2/mm²	6/mm²
Tumor-infiltrating lymphocytes	Absent	Absent
Lymphovascular invasion	Not identified	Not identified
Perineural invasion	Not identified	Not identified
Satellitosis	Not identified	Not identified
Regression / residual nevus	Not identified	Not identified
American Joint Committee on Cancer (8^th^ edition) [10]	pT4b	pT4b

Laboratory studies were obtained on multiple occasions during outpatient evaluation and hospitalization; therefore, representative results are presented as ranges reflecting longitudinal testing rather than a single time-point. Overall, there was no persistent pattern suggesting a systemic alternative explanation for the chronic plantar lesion (e.g., no sustained leukocytosis, no consistent renal or hepatic dysfunction, and preserved platelet counts). During hospitalization, inflammatory markers were elevated, including C-reactive protein (CRP) and erythrocyte sedimentation rate (ESR). The patient developed hyponatremia during inpatient care, which was clinically interpreted as compatible with syndrome of inappropriate antidiuretic hormone secretion (SIADH) in the hospital setting and managed conservatively without neurological complications. Representative findings are summarized in Table 2.

**Table 2:** Representative laboratory findings obtained longitudinally during evaluation and hospitalization. Values are shown as ranges because laboratory studies were obtained at multiple time points during outpatient evaluation and hospitalization. Overall, the results did not demonstrate a persistent renal, hepatic, hematologic, or metabolic abnormality explaining the local lesion; inflammatory markers were elevated during hospitalization, and hyponatremia was clinically interpreted as compatible with the syndrome of inappropriate antidiuretic hormone secretion.

Domain	Parameter	Observed values (range)	Reference range (as reported)	Comment
Hematology	Hemoglobin	8.7–12.5 g/dL	~10.9–15.5 g/dL (varied by report)	Mild–moderate anemia during hospitalization, improved later/other draws
Hematology	Leukocytes	8.38–10.60 ×10³/µL	4.0–11/12 ×10³/µL	No marked leukocytosis
Hematology	Platelets	262–417 ×10³/µL	150–450 ×10³/µL	Preserved platelets
Renal	Creatinine	0.60–0.75 mg/dL	0.5–0.9 mg/dL	Preserved renal function
Inflammation	C-reactive protein	40.7–44.0 mg/L	0–5 mg/L	Elevated during hospitalization (nonspecific)
Inflammation	Erythrocyte sedimentation rate	77 mm/h	2–15 mm/h	Elevated
Electrolytes	Sodium	119.8–134.2 mEq/L	135–145 mEq/L	Hyponatremia during hospitalization; near-normal pre-hospital value observed
Electrolytes	Potassium	3.69–4.94 mEq/L	3.5–5.0 mEq/L	Within reference range
Metabolic	Glucose	113–119 mg/dL	74–106 mg/dL	Mild hyperglycemia on some draws
Metabolic	HbA1c	6.90%	4.8–5.9%	Suggests relatively controlled diabetes

A staging work-up (as documented in the clinical record) included contrast-enhanced computed tomography of the thorax, abdomen, and pelvis without macroscopic distant disease, and regional nodal ultrasonography of the inguinal and popliteal basins without suspicious lymphadenopathy.

Given the patient’s advanced age, comorbidities, functional context, and the absence of macroscopic metastatic disease on staging, management after excision was oriented toward conservative/observational follow-up rather than aggressive escalation.

In the weeks following reconstruction, the postoperative course was complicated by partial graft loss, requiring advanced wound care. The wound subsequently appeared as a large postoperative plantar ulcer with granulation tissue and fibrinous slough (Figure 1E). With continued dressings and wound care, the area later demonstrated partial re-epithelialization with residual erythema, superficial crusting, and focal erosions (Figure 1F). No clinical documentation of metastatic progression was recorded during 12 months of follow-up, and conservative management was maintained. The overall chronological sequence of clinical events is summarized in Table 3.

**Table 3:** Chronological timeline summarizing the clinical evolution, diagnostic work-up, therapeutic interventions, and follow-up of the plantar acral melanoma.

Relative Time	Clinical Event
Initial presentation	Small plantar ulcerative lesion initially interpreted as a diabetic foot ulcer; local wound care started
Following months	Progressive enlargement with persistent non-healing ulceration
Subsequent progression	Development of nodular/exophytic component raises concern for neoplasm
Thereafter	Incisional biopsy performed
Histopathology result	Ulcerated acral melanoma diagnosed
Staging phase	Imaging and regional nodal assessment without evident distant disease or suspicious lymphadenopathy
Surgical management	Wide excision with graft reconstruction
Follow-up	Conservative wound management; no documented clinical progression at 12 months

## Discussion

This case illustrates a recurrent diagnostic pitfall: plantar melanoma can masquerade as a chronic diabetic foot ulcer, especially when clinicians anchor on neuropathic/pressure-related ulceration, and the lesion is treated with wound care alone [4-7]. The literature consistently shows that malignant melanoma may be initially labeled as a diabetic ulcer and that diagnosis is often delayed until atypical features emerge or healing fails despite appropriate measures [4-7]. Importantly, the systematic review of malignancies misdiagnosed as diabetic foot ulcers emphasizes that ulcerated skin cancers (including melanoma) belong in the differential diagnosis of refractory ulcers and supports a low threshold for biopsy in lesions that are atypical or non-healing [8].

Acral melanoma may present as a pigmented macule/patch with irregular borders and color variegation on the sole, but presentations are heterogeneous and can include ulceration, nodularity, exophytic growth, bleeding, fissuring, or a largely erythematous/amelanotic lesion that resembles inflammatory dermatoses or traumatic wounds [1-3]. These atypical patterns are particularly relevant on weight-bearing plantar skin, where hyperkeratosis, callus formation, secondary infection, or chronic maceration can obscure classic melanoma clues [1-3]. In the diabetic-foot context, additional confounders such as neuropathy, altered pain perception, microvascular disease, and recurrent pressure injury can further normalize the presence of a chronic plantar lesion and reduce the perceived urgency to obtain tissue diagnosis [8].

From a pragmatic standpoint, the combined message across reviews and case-based evidence is consistent: clinicians should escalate to biopsy (or urgent dermatology/surgery referral) when a “diabetic ulcer” (i) fails to improve despite adequate off-loading and wound care; (ii) progressively enlarges; (iii) develops an exophytic/nodular component; (iv) demonstrates focal pigmentation or irregular pigmentation at the margins; (v) bleeds easily; or (vi) has an atypical location/pattern for a neuropathic ulcer [1-3,8]. This approach is reinforced by the observation in our case that the lesion evolved toward an exophytic neoplastic-appearing morphology before biopsy established the diagnosis.

Beyond the clinical challenge, acral melanoma is increasingly recognized as biologically distinct from sun-exposed cutaneous melanoma. Unlike typical ultraviolet (UV)-driven melanomas, often characterized by a high burden of ultraviolet-signature point mutations, acral melanoma more commonly shows prominent chromosomal instability, structural variation, and recurrent copy-number alterations, with a comparatively lower overall point-mutation burden [11-14]. Integrative and targeted genomic studies have described recurrent changes affecting drivers of the mitogen-activated protein kinase pathway (MAPK), including alterations in the B-Raf proto-oncogene, serine/threonine kinase (BRAF); neuroblastoma RAS viral oncogene homolog (NRAS); and KIT proto-oncogene receptor tyrosine kinase (KIT) in subsets, alongside frequent focal amplifications involving cell-cycle and growth regulation (e.g., cyclin D1 (CCND1) and cyclin-dependent kinase 4 (CDK4) and other recurrent genomic events [12,13]. Whole-genome and evolutionary analyses further support that structural rearrangements and focal amplifications are major features shaping acral melanoma development and progression [14]. Clinically, this distinct genomic architecture may partially explain why acral melanoma can progress with less conspicuous early visual cues and still present with substantial thickness at diagnosis [2,3,11].

Whether diabetes specifically increases the incidence of acral melanoma remains uncertain; current evidence does not support a definitive diabetes-related predisposition to acral melanoma as a distinct entity. However, diabetes has been evaluated in relation to melanoma overall. A recent systematic review/meta-analysis found no clear association between diabetes and melanoma incidence but did find that diabetes was associated with features of more advanced disease at diagnosis (greater thickness and ulceration), consistent with delayed detection and/or differences in host or healthcare factors [15]. Earlier meta-analytic evidence suggested at most a modest increased melanoma risk among individuals with type 2 diabetes, with heterogeneity across cohorts and adjustment sets [16]. Taken together, these findings align with a clinically actionable interpretation relevant to this case: diabetes may not reliably predict melanoma risk, but it can increase the likelihood that a melanoma is discovered later, particularly when a lesion is managed under a chronic-wound paradigm rather than evaluated as a potential malignancy [8,15].

In our case, the discrepancy between incisional and excisional depth (Table 1) underscores that limited sampling can underestimate tumor burden in plantar lesions. While biopsy remains essential for diagnosis, clinicians should remain aware that ulcerated acral lesions can be heterogeneous and that definitive excision may provide a more accurate assessment of invasion and other high-risk features [2,3]. Finally, management in very elderly patients should be individualized; reviews addressing acral melanoma emphasize balancing oncologic benefit with comorbidity burden, frailty, functional status, and goals of care, particularly when staging does not show macroscopic metastatic disease (as in this case per clinical record) [9].

## Conclusions

Plantar melanoma can mimic diabetic foot ulceration in older adults with diabetes, potentially leading to diagnostic delay when lesions are attributed to chronic ulceration. Clinicians should consider early biopsy or specialist referral for plantar lesions that fail to heal as expected or that develop atypical clinical features. Prompt recognition of malignant mimics is essential to avoid prolonged mismanagement and to enable appropriate staging and treatment decisions.
